# Anti-Oncogenic *gem*-Dihydroperoxides Induce Apoptosis in Cancer Cells by Trapping Reactive Oxygen Species

**DOI:** 10.3390/ijms17010071

**Published:** 2016-01-08

**Authors:** Yuki Kuranaga, Nami Yamada, Maiko Kashiwaya, Moeko Nakamura, Lei Cui, Minami Kumazaki, Haruka Shinohara, Nobuhiko Sugito, Kohei Taniguchi, Yuko Ito, Tatsushi Nakayama, Bunji Uno, Akichika Itoh, Yukihiro Akao

**Affiliations:** 1 United Graduate School of Drug Discovery and Information Science, Gifu University, 1-1 Yanagido, Gifu 501-1193, Japan; t3125010@edu.gifu-u.ac.jp (Y.K.); namiyamada80@gmail.com (N.Y.); t3501001@edu.gifu-u.ac.jp (M.K.); t3501002@edu.gifu-u.ac.jp (H.S.); t3125016@edu.gifu-u.ac.jp (N.S.); sur144@poh.osaka-med.ac.jp (K.T.); 2 Graduate School of Engineering, Gifu University, 1-1 Yanagido, Gifu 501-1193, Japan; 3 Department of Pharmacy, Gifu Pharmaceutical University, 1-25-4, Daigaku-nishi, Gifu 501-1196, Japan; vo.ov.happy@gmail.com (M.K.); 106028@gifu-pu.ac.jp (M.N.); Cui.Lei@otsuka.jp (L.C.); tnakayama@gifu-pu.ac.jp (T.N.); uno@gifu-pu.ac.jp (B.U.); itoha@gifu-pu.ac.jp (A.I.); 4 Department of Anatomy and Cell Biology, Division of Life Sciences, Osaka Medical College, 2-7 Daigaku-machi, Takatsuki, Osaka 569-8686, Japan; an1006@art.osaka-med.ac.jp

**Keywords:** apoptosis, ROS, dihydroperoxide, JNK/MAPK, cancer cell

## Abstract

Organic *gem*-dihydroperoxides (DHPs) and their derived peroxides have attracted a great deal of attention as potential anti-cancer agents. However, the precise mechanism of their inhibitory effect on tumors is unknown. To determine the mechanism of the inhibitory effects of DHPs, we examined the effects of DHPs on leukemia K562 cells. As a result, certain DHPs used in this study exhibited growth-inhibitory activity according to a clear structure-activity relationship. The most potent DHP, 12AC3O, induced apoptosis in K562 cells, but not in peripheral blood monocytes (PBMCs) or fibroblast cells. 12AC3O induced apoptosis through the intrinsic mitochondrial pathway and thereafter through the extrinsic pathway. The activity of the former pathway was partly attenuated by a JNK inhibitor. Interestingly, 12AC3O induced apoptosis by trapping a large amount of ROS, leading to an extremely lower intracellular ROS level compared with that in the cells in the steady-state condition. These results suggest that an appropriate level of intracellular ROS was necessary for the maintenance of cancer cell growth. DHPs may have a potential to be a novel anti-cancer agent with minimum adverse effects on normal cells.

## 1. Introduction

**Figure 1:**
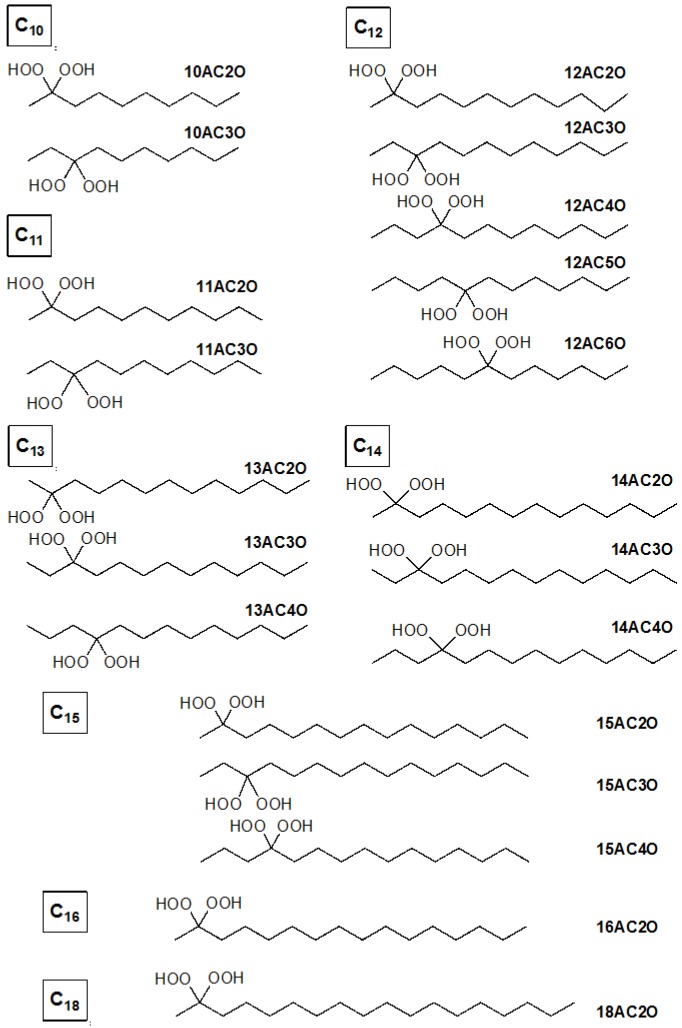
Dihydroperoxide (DHP) agents used in this study.

Reactive oxygen species (ROS) are always generated in the human body as a consequence of respiration and oxygen metabolism, but their accumulation can cause oncogenic damage to cells and tissues [1]. ROS are the most important redox signaling molecules, and are involved in the regulation of the MAPK signaling pathway, which induces the death of cancer cells through accumulating ROS. These pleiotropic effects of ROS are largely accounted for by changes in the thiol/disulfide status of the cell, an important determinant of the cell’s redox status [1]. Hydrogen peroxide, a ROS, also induces apoptotic and necrotic cell death through the induction of oxidative stress in cells [2,3]. In this context, we focused our attention on the effect of the peroxy group; and so we synthesized novel *gem*-dihydroperoxides (DHPs), which can be easily prepared from commercially available compounds (Figure 1) [4,5]. DHPs and their derived peroxides are known to have anti-malarial and -cancer effects [6,7,8]. At first, we assumed that the DHPs may induce cancer cell death more effectively than peroxide compounds by causing the accumulation of ROS in the cells due to their double peroxy groups. In the current study, a structure-activity relationship of the cell growth inhibitory effect on leukemia cells was found. Among the DHPs examined, 12AC3O most efficiently induced apoptosis. Unexpectedly, the mechanism of apoptotic cell death was due to deprivation of ROS in cancer cells; and the apoptotic cell death occurred at least in part through the activation of JNK/MAPK, a stress-activated protein kinase [9]. 12AC3O-induced apoptosis was mainly due to a decreased ROS level, but the above-mentioned stress-inducing signaling cascade was also induced as in the case of excessive ROS. These results suggest the essential roles of ROS in the maintenance of growth-related signaling pathways. In addition, 12AC3O and other DHPs had no effect on cell growth in concanavalin-A stimulated peripheral blood monocytes (PBMCs). 12AC3O may thus be useful not only as an anti-cancer agent but also as an agent for discrimination between cancer and normal cells, and for the scavenging of free radicals.

## 2. Results

### 2.1. Effects of DHPs on Cell Growth in Human Leukemia K562 Cells

**Figure 2:**
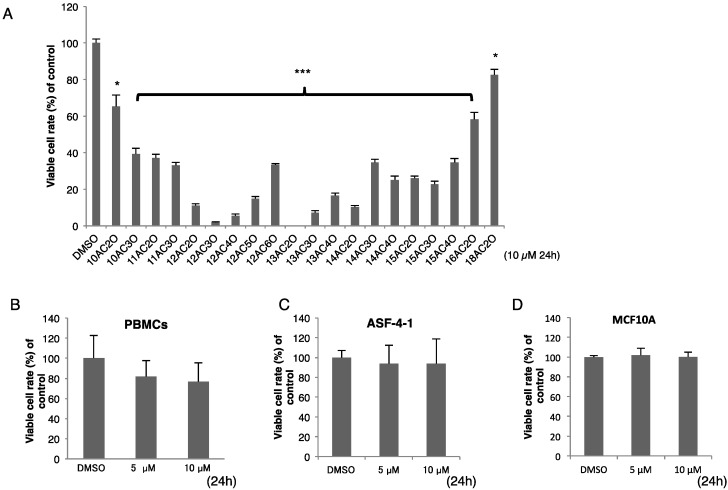
DHPs inhibited cell proliferation of K562 human leukemia cells but had no effect on human peripheral lymphocytes or other normal human cells. (**A**) Human leukemia K562 cells were treated with DHPs (10 µM) for 24 h. Viable cells were evaluated by using trypan-blue staining. DMSO was used as a control. *t*-test * *p* < 0.05, *** *p* < 0.001 *versus* the control; (**B**–**D**) Human peripheral blood monocytes (PBMCs, **B**), human normal diploid fibroblast ASF-4-1 cells (**C**), and human normal mammary epithelial cells, MCF10A (**D**), were treated for 24 h with 12AC3O (5, 10 µM). DHP 12AC3O had the most potent growth inhibitory activity.

Firstly, we examined whether DHPs exhibited a growth inhibitory effect on human leukemia K562 cells. As shown in Figure 2A, various kinds of DHPs showed anti-cancer activity toward K562 cells at 24 h after the start of treatment. Especially, in the C-12 series, the activity was shown to be dependent on the position of the diperoxide group in the structure. Interestingly, a structure-activity relationship was found: growth inhibition was greater when the number of C atoms was approximately from 11 to 15 and the position of the diperoxide group was approximately from 2 to 4. 12AC3O showed the greatest anti-cancer activity among the DHPs, but it did not show any effect on the growth of human concanavalin-A-stimulated peripheral lymphocytes, ASF-4-1 human skin fibroblasts or MCF-10A human normal mammary epithelial cells at 24 h after the start of treatment (Figure 2B–D). The same structure-activity relationship was also observed for cells of the human colorectal cancer cell line DLD-1 (data not shown). These results indicated that DHPs exhibited their growth-inhibiting effect specifically on cancer cells. Based on the IC_50_ of the DHPs tested and their stability, we chose 12AC3O for use in subsequent experiments. Interestingly, its IC_50_ value was lower than that of leukemia therapeutic agents such as Etoposide and Cytarabine (Table 1) [10].

**Table 1:** IC_50_ values for the C-12 series of DHPs (using K562 cells).

Agent	IC_50_ Value (µM)
12AC2O	1.65
12AC3O	0.81
12AC4O	1.69
12AC5O	2.14
12AC6O	3.83
Etoposide	5.30
Cytarabine	2.10

Viable cell ratio of C-12 series of DHP or leukemia therapeutic agent-treated K562 cells at 24.

### 2.2. 12AC3O Induced Apoptotic Cell Death

**Figure 3:**
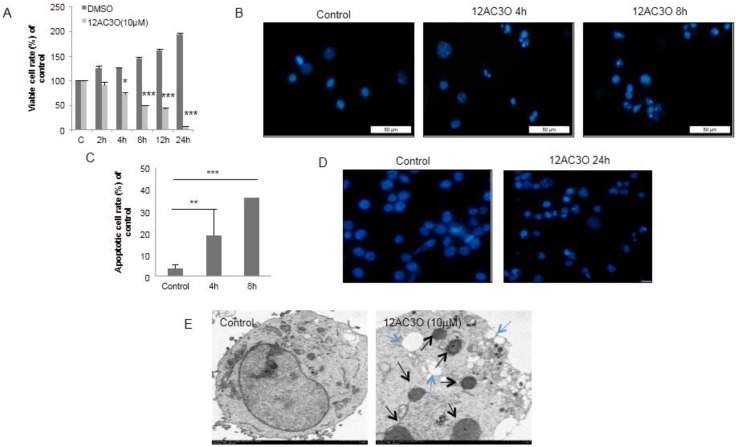
12AC3O induced apoptosis in K562 cells as estimated by morphological examination. (**A**) Viable cell ratio of 12AC3O (10 µM)-treated K562 cells up to 24 h after the start of treatment. The viable cells were counted over time by using trypan-blue staining. *t*-test * *p* < 0.05, *** *p* < 0.001 *versus* the control; (**B**,**C**) The morphological characteristics of apoptosis in K562 cells were observed by fluorescence microscopy using Hoechst 33342 (5 µg/µL). Bar is 50 µm. *t*-test ** *p* < 0.01, *** *p* < 0.001 *versus* the control; (**D**) Morphological characteristics of apoptosis in K562 cells observed by electron microscopy; (**E**) The black arrows indicate fragmented nuclei; and the blue arrows, autophagosomes.

12AC3O induced growth inhibition of K562 cells time-dependently (Figure 3A). In order to examine how the growth of 12AC3O-treated cells was inhibited, we stained the treated cells with Hoechst 33342 and found some apoptotic cells with characteristic chromatin condensation and fragmentation at 4 h after the start of treatment (Figure 3B). The number of apoptotic cells increased in a time-dependent manner (Figure 3C). In order to confirm whether 12AC3O could induce apoptotic cell death in other cancer cell lines, we treated Jurkat T-cell leukemia cells and DLD-1 colorectal cancer cells with 12AC3O and then performed a viable cell assay by using the Trypan-blue dye-exclusion test and Hoechst 33342 staining. As a result, 12AC3O also induced growth inhibition and apoptotic chromatin condensation and fragmentation in these cells (Figure S1A–C). Morphologically, the EM study also indicated the apoptotic fragmentation of the nucleus in the treated-K562 cells (Figure 3E, black arrows). In some of the cells, autophagosomes were observed (blue arrows). Thus, 12AC3O induced mainly apoptosis, and in part autophagy, in the early phase; however, later apoptotic cell death dominated.

**Figure 4:**
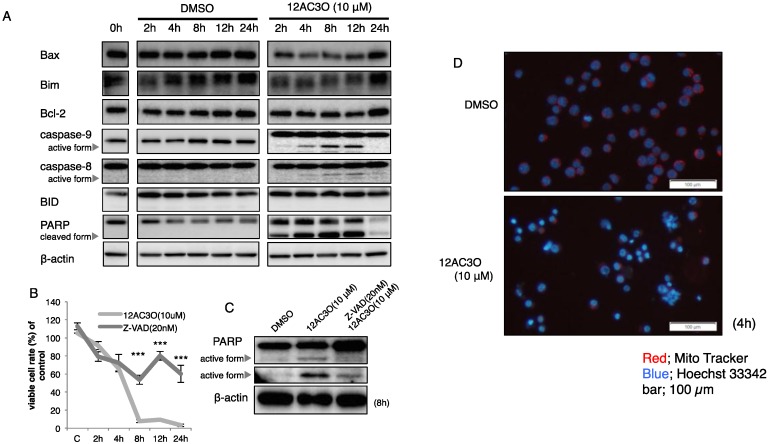
Profile of intracellular signaling pathways in 12AC3O-treated K562 cells. (**A**) Expression of apoptosis-related proteins after treatment with 12AC3O (10 µM) for 2, 4, 8, 12 or 24 h, as assessed by Western blot analysis; (**B**) Viable cell ratios of 12AC3O-treated (10 µM) K562 cells at 2, 4, 8, 12 or 24 h after pre-treatment with Z-VAD, a caspase inhibitor, for 24 h; (**C**) Change in protein expression profiles of PARP and its cleaved form. *t*-test *** *p* < 0.001 12AC3O-treated *versus* 12AC3O-treated K562 cells in the presence of Z-VAD; (**D**) Measurement of mitochondrial membrane potential in K562 cells after treatment with 12AC3O (10 µM) at 4 h by using Mito-tracker. Blue fluorescence indicates positive Hoechst 33342 nuclear staining.

Next, we surveyed the expression of signaling proteins associated with apoptosis by performing Western blotting. As a result, the activation of caspase-8 continued from 4 to 24 h after the start of treatment. The cleaved-forms of PARP were observed from 2 h up to 12 h after treatment; and caspase-9 was also activated at the time corresponding to the cleavage of PARP (Figure 4A). However, the expression levels of pro-apoptotic Bax and Bim remained almost unchanged. At 24 h after the start of treatment, some cells that were not sensitive to 12AC3O continued to proliferate. The expression level of Bcl-2 became elevated at 24 h. The expression level of BID remained unchanged, and its truncated form couldn’t be detected (Figure 4A). In order to further certify that 12AC3O induced apoptosis, we used the caspase inhibitor Z-VAD (MBL). Pre-incubation with Z-VAD clearly inhibited the increase in the number of apoptotic K562 cells after treatment of them with 12AC3O (Figure 4B). Biochemically, the bands of cleaved-form PARP demonstrated by the treatment with 12AC3O were significantly attenuated by the pre-treatment with Z-VAD (Figure 4C). Next, we examined the mitochondrial membrane potential by staining the cells with Mito-Tracker. In Figure 4D, the bottom photo shows that the mitochondrial membrane potential was clearly decreased in the cells after treatment with 12AC3O at 4 h compared with that after treatment with DMSO as the control. These findings taken together indicate that 12AC3O induced apoptotic cell death mainly through the intrinsic apoptotic signal pathway and that the extrinsic apoptotic signal pathway was subsequently activated to execute apoptosis completely.

### 2.3. The SAPK/JNK Was Up-Regulated in the Early Phase of Treatment with 12AC3O

**Figure 5:**
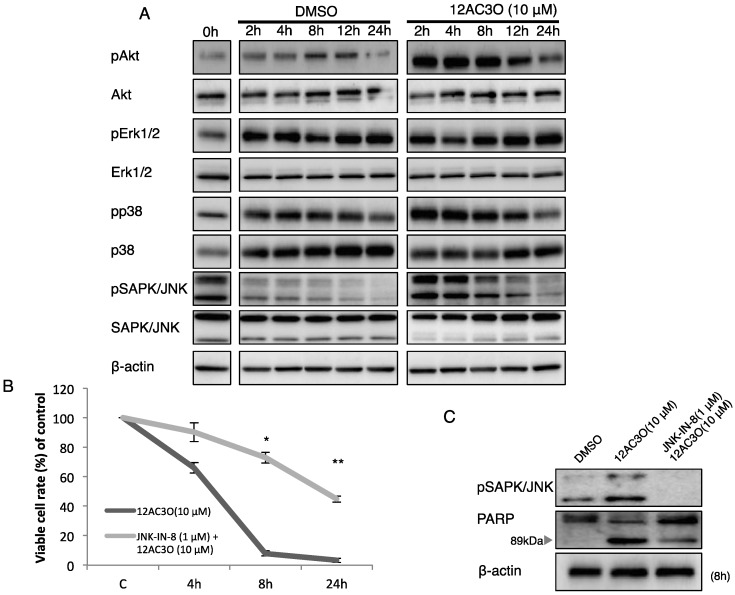
Growth-related signaling pathways in 12AC3O-treated K562 cells. (**A**) Time-dependent protein expression profiles of growth-related signaling of MAPK and PI3K/Akt in 12AC3O-treated K562 cells. DMSO was used as a control; (**B**) Viable cell ratio of 12AC3O-treated K562 cells at 4, 8, and 24 h after pre-treatment with JNK-IN-8, a JNK inhibitor, for 24 h; (**C**) Change in the protein expression profiles of PARP and its cleaved form. *t*-test * *p* < 0.05, ** *p* < 0.01, 12AC3O-treated *versus* 12AC3O-treated K562 cells in the presence of JNK-IN-8.

To understand the effect of 12AC3O on MAP kinases and the growth-related PI3K/Akt signaling pathway in the treated K562 cells, we performed Western blotting analysis. As to MAP kinases, pErk/Erk was weakly activated; however, pp38/p38 and pJNK/JNK were activated until 4 h and then became gradually inactive from 8 h after the start of treatment (Figure 5A). Next, we pre-treated cells with the JNK-IN-8 JNK inhibitor (EMD Chemicals) in order to validate the role of JNK in 12AC3O induced-apoptosis. Interestingly, the apoptotic cell death was significantly suppressed by the treatment with JNK-IN-8 at 1 µM (Figure 5B), which reflected the decreased level of cleaved-form PARP. In contrast, the level of PARP was increased (Figure 5C). These findings indicate that JNK played a key role in the apoptosis induced by 12AC3O. The PI3K/Akt signaling pathway was activated until 8 h and inactivated on 8 h up to 24 h, which could reflect a compensatory survival signaling against 12AC3O.

### 2.4. Superoxide-Scavenging Effect of 12AC3O on K562 Cells

**Figure 6:**
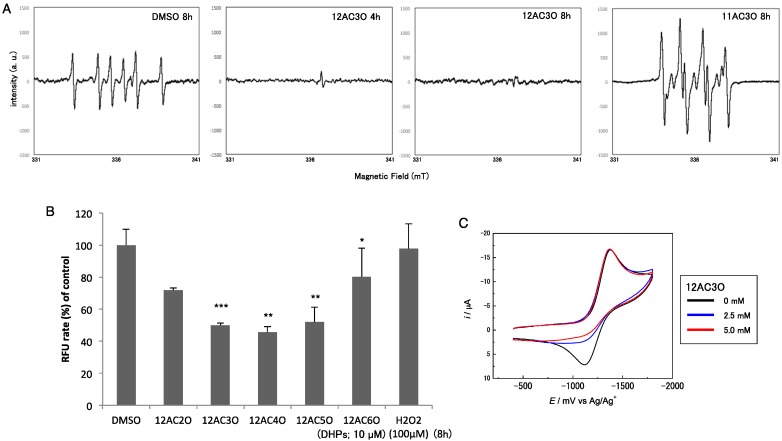
ROS-scavenging effect of DHPs. (**A**) ESR spectra of the sample solutions treated with DMSO (control) for 8 h, 12AC3O for 4 and 8 h or 11AC3O for 8 h. The sample preparation was described in the Materials and Methods section; (**B**) Intracellular ROS levels in K562 cells treated with the C-12 series of DHPs, as estimated by performing the intracellular ROS assay. DMSO was used as a control. *T*-test * *p* < 0.05, ** *p* < 0.01, *** *p* < 0.001 *versus* DMSO control; (**C**) Cyclic voltammograms of O_2_ in the absence and in the presence of 12AC3O in DMF containing 0.1 M tetrabutylammonium perchlorate on a GC electrode. The scan rate was 1 V/s. Concentrations of 12AC3O are 0 (black line), 2.5 (blue line), and 5.0 mM (red line).

Based on the features of DHP structures, we conducted additional experiments to obtain direct evidence that 12AC3O scavenged superoxide (O_2_·−) or hydroperoxy (HO_2_·) radicals derived from O_2_·− by protonation. The intracellular ROS level in human leukemia K562 cells after treatment with DHPs was examined by making Electron Spin Resonance (ESR) spectral measurements using the spin-trap technique with 5,5-dimethyl-1-pyrroline-*N*-oxide (DMPO). In the absence of 12AC3O, the ESR spectrum of the DMPO adduct of O_2_·− or HO_2_· was obtained, as shown in Figure 6A. Fitting simulation of the ESR spectrum gave hyperfine coupling constants of 1.59 mT for N and 2.29 mT for H, showing generation of the DMPO adduct. However, in K562 cells treated with 10 µM 12AC3O for 4 h, only a slight generation of the DMPO adduct was detected; and after 8 h of treatment no ESR response was obtained, indicating no trapping by DMPO. In K562 cells treated with 10 µM 11AC3O for 8 h, which DHP did not show any significant anti-cancer effect, the DMPO adduct was definitely detected (Figure 6A). These results indicate that the O_2_·− or HO_2_· scavenging reaction with 12AC3O proceeded faster than the spin trap reaction of DMPO, suggesting deep involvement of ROS trapping in 12AC3O-induced apoptosis. We further confirmed the decrease in intracellular ROS by using an OxiSelect Intracellular ROS Assay Kit (CELL BIOLABS), which results were similar to those obtained by ESR in 12AC3O-treated cells (Figure 6B).

Chemical evidence for the ROS-scavenging capacity of 12AC3O was also obtained by making electrochemical measurements. Figure 6C shows the cyclic voltammograms of the O_2_/O_2_·− redox couple in the absence and in the presence of 12AC3O. In the case of O_2_ reduction in aprotic solvents such as dimethylformamide (DMF), the cathodic peak corresponding to the generation of O_2_·− was followed in the subsequent anodic scan by a well-defined oxidation peak associated with regeneration of the starting materials (O_2_), showing the reversible redox wave (Black line, Figure 6C). It is well documented that the presence of acidic substances during O_2_ reduction induces a change from a reversible monoelectronic process to O_2_·− to an irreversible bielectronic process to H_2_O_2_, driven by the exergonic reduction of HO_2_· generated after the primary electrode process associated with proton transfer from the acid [11,12]. However, the presence of 12AC3O was only associated with an apparent decrease in the reversibility of the O_2_/O_2_·− redox couple, as shown in Figure 6C. The monoelectronic waves with the disappearance of the reoxidation wave (Blue and Red lines in Figure 6C) are typical of voltammograms showing that HO_2_· produced during the electrochemical process in the presence of 12AC3O was consumed immediately by the proton-coupled electron transfer from 12AC3O, without being reduced on the electrode [13]. Therefore, our data strongly suggest that the peroxide moiety in 12AC3O played an important role in scavenging O_2_·− by direct electron and proton transfer from 12AC3O. Thus, the apoptosis induced by 12AC3O could have been due to a large reduction in the amount of intracellular ROS, which contributes to maintenance of the growth-related signaling pathways.

### 2.5. 12AC3O Inhibited Tumor Progression in Vitro

**Figure 7:**
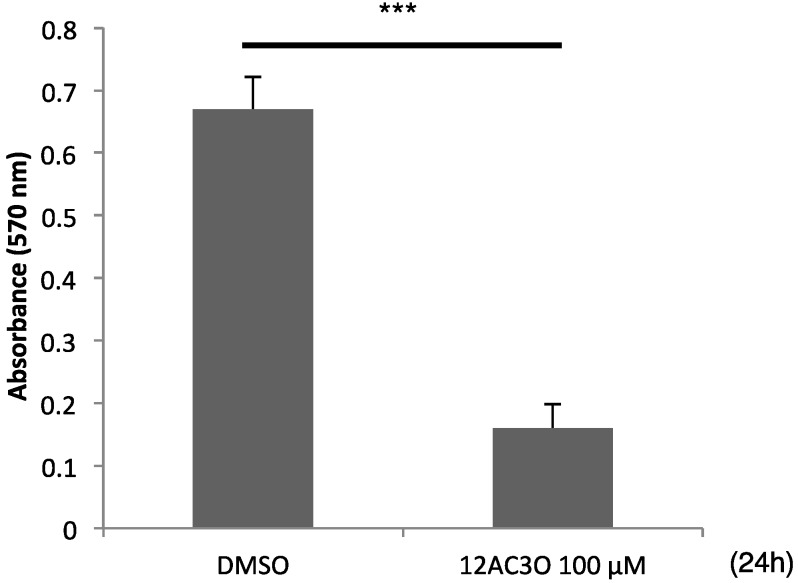
Anti-tumor effects of 12AC3O estimated by using DLD-1 cells in the 3D spheroid assay. The cell viability of tumor cells within the spheroids was measured by using the MTT assay. *t*-test *** *p* < 0.001 *versus* DMSO control.

In order to examine whether 12AC3O could suppress the growth of solid tumors, we used the 3D spheroid system instead of performing an *in vivo* experiment using mice. As a result, although the spheroids of DLD-1 cells exhibited a rapid growth in the presence of DMSO, the growth of the tumor cells was significantly suppressed by treatment with 12AC3O at 100 µM (Figure 7A). These data thus reflected the growth inhibition of spheroids treated with 12AC3O *in vitro.*

## 3. Discussion

Our goal was to determine the precise mechanism of the tumor-inhibitory effect of DHPs. Our results suggest that one of the most potent DHPs, 12AC3O, induced apoptosis only in cancer cells and did so through trapping a large amount of ROS, as was confirmed by using ESR spectrometry, intracellular ROS assay, and cyclic voltammetry. It is well known that the accumulation of intracellular ROS leads to apoptotic cell death of cancer cells [14,15,16]. Responsible signaling pathways involved in ROS-inducing cell death have been identified as JNK and p38 MAPK pathways [17,18,19]. However, it has been also reported that cancer cells require a small amount of ROS to stimulate growth signaling and proliferation [20,21]. In the current study, DHPs activated JNK and p38 signaling and induced apoptosis in cancer cells through the depletion of their intracellular ROS. Therefore, our data indicate that extremely diminished ROS levels caused by DHPs could stimulate apoptotic signaling in cancer cells. Based on our biochemical experiments, apoptotic cell death from 8 up to 12 h was shown from the results of Western blotting analysis. On the other hand, bcl-2 tended to be down-regulated in its expression at 12 h after treatment with 12AC3O, but up-regulated at 24 h, reflecting the growth of the cells escaping from apoptotic cell death.

We also presented data showing that apoptosis induction by DHPs occurred only in cancer cells and not in normal cells such as PBMCs, fibroblasts, and epithelial cells. Most cancer cells rely on aerobic glycolysis, termed “the Warburg effect”, which generates minimum ROS within the cells [22,23]. However, as mentioned above, an adequate amount of ROS is needed in cancer cells for them to grow [20,21]. DHPs deprived cancer cells of almost all their ROS, whereas depletion of ROS from normal cells led to a level that was still within the tolerable range. Therefore, we consider that the cancer cells were much more sensitive to the DHPs than normal cells.

In summary, our work reveals that ROS produced in steady-state growth is needed to maintain growth and survival of cancer cells. More studies will be needed to clarify the role of ROS in growth signaling pathways as well as the difference in activated signaling pathways between cancer and normal cells.

In the future, 12AC3O may become a new free radical scavenger drug, inhibiting oxidative injury and protecting against ischemic diseases such as cardiac infarction and cerebral infarction.

## 4. Materials and Methods

### 4.1. Synthesis of Germinal (gem)-Dihydroperoxides

*gem*-dihydroperoxides were synthesized from their corresponding ketones through dihydroperoxidation with H_2_O_2_ or O_2_ in the presence of catalytic iodine or photosensitizer as mentioned in previous reports [4,5]. *gem*-dihydroperoxides were easily obtained from the corresponding carbonyl compounds in high yields through a catalyst-free method using aqueous H_2_O_2_ (35%) in 1,2-dimethoxyethane at room temperature [4].

### 4.2. Cell Culture and Cell Viability

Human leukemia cell line K562, Jurkat T-cell leukemia, and human colorectal cancer cell line DLD-1 were maintained in RPMI-1640 medium (Wako Pure Chemical Industries, Ltd., Osaka, Japan) supplemented with 8% (*v*/*v*) heat-inactivated FBS (Sigma–Aldrich Co., St. Louis, MO, USA) and 2 mM l-glutamine. Human diploid fibroblast cell line ASF-4-1 was maintained in E-MEM medium (Wako) supplemented with 10% (*v*/*v*) heat-inactivated FBS (Sigma–Aldrich Co.) and 2 mM l-glutamine. Mammary gland epithelial cell line MCF10A cells were maintained in MEBM medium (Lonza, Tokyo, Japan). These cell lines were cultured under an atmosphere of 95% air and 5% CO_2_ at 37 °C. The number of viable cells was determined by performing the trypan-blue dye exclusion test. Each diperoxide tested was dissolved in DMSO. In control experiments the cells were incubated with DMSO alone.

### 4.3. Purification of Peripheral Blood Monocytes (PBMCs)

Human peripheral blood samples were obtained from healthy donors with informed consent of the subjects and following ethical approval. PBMCs were purified by density-gradient centrifugation using Ficoll-Paque PLUS (GE Healthcare Bio-Sciences, Uppsala, Sweden). First, a blood sample was layered onto Ficoll-Paque PULS in a centrifuge tube, which was then centrifuged at 400× *g* for 30 min. Then the lymphocytes layer was collected and washed to remove the platelets. The PBMCs thus collected were re-suspended at 10^6^ cells/ml in RPMI-1640.

### 4.4. Morphological Assessment of Apoptosis

For assessment of the morphological characteristics of apoptosis, the cells were stained with Hoechst 33342 (5 µg/mL) at 37 °C for 30 min, washed once with phosphate-buffered saline (PBS), resuspended in PBS, dropped onto a glass slide, and examined by fluorescence microscopy using an Olympus fluorescence microscope (Tokyo, Japan) equipped with an epi-illuminator and appropriate filters. The cells with condensed and fragmented nuclei stained with Hoechst 33342 were taken to be apoptotic. The mitochondrial membrane potential was assessed by use of a fluorescent dye, Mito-Tracker Orange (Molecular Probes, Eugene, OR, USA), which accumulates selectively in active mitochondria and becomes fluorescent when oxidized [24] Mito-Tracker Orange was added to the culture medium at a concentration of 10 nM. After the cells had been treated with Mito-Tracker Orange and washed with PBS, they were resuspended in PBS and observed under the fluorescence microscope.

### 4.5. Inhibitor Agents

In order to confirm the induction of apoptosis by 12AC3O, we used a pan-caspase inhibitor, Z-VAD-fmk (zVal-Ala-Asp-fluoromethyl ketone), which was purchased from MBL (Nagoya, Japan) and a JNK inhibitor, JNK-IN-8, from EMD chemicals (San Diego, CA, USA). These inhibitors were added to the culture medium 24 h before treatment with 12AC3O. The optimal concentration of the caspase inhibitor and JNK inhibitor to inhibit cell death was determined from a dose–response curve and was found to be 20 nM and 10 µM, respectively. Inhibition of apoptosis by the inhibitor was evaluated by measuring the number of live cells, which was determined by use of the trypan blue dye-exclusion test, and by Western blot analysis to detect the weakened level of the cleaved-form of PARP. In order to inhibit intracellular ROS, we used the ROS inhibitor *N*-acetyl-l-cysteine (NAC; Sigma–Aldrich Co.).

### 4.6. Protein Extraction and Western Blotting

The cells were homogenized in chilled lysis buffer comprising 10 nM Tris–HCl (pH 7.4), 1% NP-40, 0.1% deoxycholic acid, 0.1% SDS, 150 mM NaCl, 1 mM EDTA, and 1% Protease Inhibitor Cocktail (Sigma–Aldrich Co.) and stood for 20 min on ice. After centrifugation at 13,000 rpm for 20 min at 4 °C, the supernatants were collected as whole-cell protein samples. Protein contents were measured with a DC protein assay kit (Biorad, Hercules, CA, USA). Lysate proteins (10 µg) were separated by SDS-PAGE using a 10% or 12.5% polyacrylamide gel (Wako), and electroblotted onto a PVDF membrane (Millipore Corporation, Billerica, MA, USA). After blockage of nonspecific binding sites for 1 h with 5% nonfat milk in TBS-T, the membrane was incubated overnight at 4 °C with primary antibodies, which included those against the following antigens: PARP, cleaved caspase-8, Bid, LC3B, phospho-Akt (Ser473), Akt, phospho-Erk1/2 (Thr202/Tyr204), Erk, phospho-p38 (Thr180/Tyr182), p38, phospho-SAPK/JNK (Thr183/Thr185), SAPK/JNK (Cell Signaling Technology, Danvers, MA, USA); caspase-8, -9, Bax (MBA, Nagoya, Japan); Bim and Bcl2 (Santa Cruz Biotechnology, Santa Cruz, CA, USA), all properly diluted with TBS-T containing 2% bovine serum albumin and 0.01% sodium azide or Solution 1 of Can Get Signal Immunoreaction Enhancer Solution (TOYOBO Co., Ltd., Osaka, Japan). The membrane was then washed 3 times with TBS-T, incubated further with HRP-conjugated horse anti-mouse or goat anti-rabbit IgG antibody (Cell Signaling Technology) for 1 h at room temperature, and then again washed 3 times with TBS-T. The immunoblots were visualized by use of Luminata™ Forte Western HRP Substrate (Millipore Corporation). The quantity loaded was verified by re-incubating the same membrane with anti-β-actin antibody (Sigma–Aldrich Co.).

### 4.7. Electron Microscopic Study

K562 cells treated with DMSO or 12AC3O (10 µM) at 24 h were rinsed with PBS. The cells were then fixed for 2 h with 2% paraformaldehyde and 2.5% glutaraldehyde in 0.2 M phosphate buffer (pH 7.4, PB), rinsed in PB, and postfixed in 2% osmium tetraoxide for 2 h. After having been washed with PB, the cells were progressively dehydrated by passage through a 10% graded series of 30%–100% ethanol and then cleared in QY-1 (Nissin EM, Tokyo, Japan). Thereafter, they were embedded in Epon 812 resin (TAAB Laboratories Equipment, Reading, UK); and thin sections (70-nm thickness) were prepared. Finally, the sections were stained with uranyl acetate and lead citrate and examined by transmission electron microscopy with a Hitachi-7650 (Hitachi, Tokyo, Japan), operating at 80 kV.

### 4.8. Electron Spin Resonance (ESR) Spectral Measurements

In order to detect free radicals in K562 cells treated with 12AC3O, we used electrochemical ESR spectrometry with a JES-FA200 X-band spectrometer (JEOL Resonance Inc., Tokyo, Japan). The spin-trap technique using 5,5-dimethyl-1-pyrroline-*N*-oxide (DMPO; Tokyo Chemical Industry Co., Ltd., Tokyo, Japan) was used for estimation of the O_2_·− scavenging of DHPs. The measurement conditions were as follow: magnetic field, 336 ± 5 mT; power, 8.98 mW; sweep time, 4 min; modulation, 100 kHz, 0.08 mT; amplitude, 4000; and time constant, 0.3 s. Samples for ESR measurements were prepared as follows: K562 cells were treated with DMSO, 10 µM 12AC3O or 10 µM 11AC3O for 4 or 8 h; and then the cells were dissolved in DMPO. ESR spectra of these samples were obtained by using a quartz tube fabricated for water samples.

### 4.9. Intracellular ROS Assay

In order to detect intracellular ROS in K562 cells treated with 12AC3O, we used an OxiSelect Intracellular ROS Assay Kit (Cell Biolabs, San Diego, CA, USA). First, K562 cells (5 × 10^5^ per well) of K562 were treated at 37 °C for 60 min with DCF dye in the cell culture medium. Then, the cells were washed twice with PBS. After that, the K562 cells were incubated with 12AC2O, 3O, 4O, 5O or 6O, each at 10 µM, in a 12-well plate at 37 °C for 8 h. After removal of the media by centrifugation and washing of the cells with PBS, the cells were next re-suspended and incubated in RPMI medium with cell lysis buffer for 5 min. Fluorescence intensities were quantified by use of a Varioskan Flash (Thermo Scientific, Waltham, MA, USA) at 480/530 nm with a 530-nm cutoff. All samples were prepared in triplicate, and data were presented as the mean ± SD from 3 experiments. Unpaired two-tail *t* tests were performed to determine significance of differences.

### 4.10. Electrochemical Measurements

Cyclic voltammetry was performed at 25 °C with a three-electrode system consisting of a glassy carbon (GC) working electrode, a coiled platinum counter electrode, and an Ag/AgNO_3_ reference electrode used for nonaqueous solvents (BAS RE-5). A BAS 100B electrochemical workstation coupled to a Dell Optiplex760 PC using BAS electrochemical software was used to record and analyze the data. The GC electrodes were polished with alumina powder (1 μm) and ultrasonically rinsed with anhydrous CH_3_CN prior to each run.

Samples for electrochemical measurements were prepared in a glove box completely filled with N_2_ gas to prevent contamination by moisture. The DMF solution containing 0.1 M TPAP as a supporting electrolyte was saturated with O_2_ by bubbling the gas for *ca.* 2−3 min, and the gas was passed over the solutions during the measurements to maintain the concentration of O_2_ at a constant level. The equilibrium concentration of O_2_ was calculated to be 4.8 mM.

### 4.11. 3D Spheroid Colorimetric Proliferation/Viability Assay

The 3D spheroid colorimetric proliferation/viability assay was performed according to the manufacturer’s protocol provided in a Cultrex 3-D spheroid Colorimetric Proliferation/Viability Assay Reagent Kit (Trevigen, Inc., Gaithersburg, MD, USA). Approximately 3000 DLD-1 cells were suspended in 1X Spheroid Formation ECM reagent. Then a 50 µL aliquot of the cell suspension was added to each well of a 3D culture Qualified 96-Well Spheroid Formation Plate. After that, the plate was centrifuged at 200× *g* for 3 min at room temperature in a swinging bucket rotor. Then, it was incubated at 37 °C in a tissue culture incubator for 72 h to promote spheroid formation. Thereafter the spheroids were treated with 12AC3O for 24 h at 37 °C. After that 5 µL of MTT Reagent was added per well, and the plate was incubated further for 24 h. Next, 55 µL of detergent reagent was added to each well, and incubation was resumed at 37 °C for 24 h to solubilize the cells and MTT formazan crystals that had formed. Twenty hours later, the absorbance was read at 570 nm.

### 4.12. Statistics

Each examination was performed in triplicate. Differences were statistically evaluated by one-way analysis of variance followed by the *t-*test. Data were presented as means ± SD. A *p* value of less than 0.05 was considered to be statistically significant.

## Supplementary Materials

Supplementary materials can be found at http://www.mdpi.com/1422-0067/17/1/71/s1.

## Abbreviations

ROS: reactive oxygen species; DHPs: *gem*-dihydroperoxides; PBMCs: peripheral blood monocytes; DMSO: dimethyl sulfoxide; ESR: electron spin resonance; DMPO: 5,5-dimethyl-1-pyrroline-*N*-oxide; DMF: dimethylformamide.
