# Supplementary material for: Anti-Oncogenic gem-Dihydroperoxides Induce Apoptosis in Cancer Cells by Trapping Reactive Oxygen Species
Source: Int J Mol Sci. 2016 Jan 8;17(1):71. doi: 10.3390/ijms17010071 (PMC4730315; doi:10.3390/ijms17010071)
Supplement: Supplementary file 1 [file ijms-17-00071-s001.pdf]

Int. J. Mol. Sci. **2016**, *17*, 71; doi:10.3390/ijms17010071

Supplementary Materials: Anti-Oncogenic *Gem*-Dihydroperoxides Induce Apoptosis in Cancer Cells by Trapping Reactive Oxygen Species

Yuki Kuranaga, Nami Yamada, Maiko Kashiwaya, Moeko Nakamura, Lei Cui, Minami Kumazaki, Haruka Shinohara, Nobuhiko Sugito, Kohei Taniguchi, Yuko Ito, Tatsushi Nakayama, Bunji Uno, Akichika Itoh and Yukihiro Akao

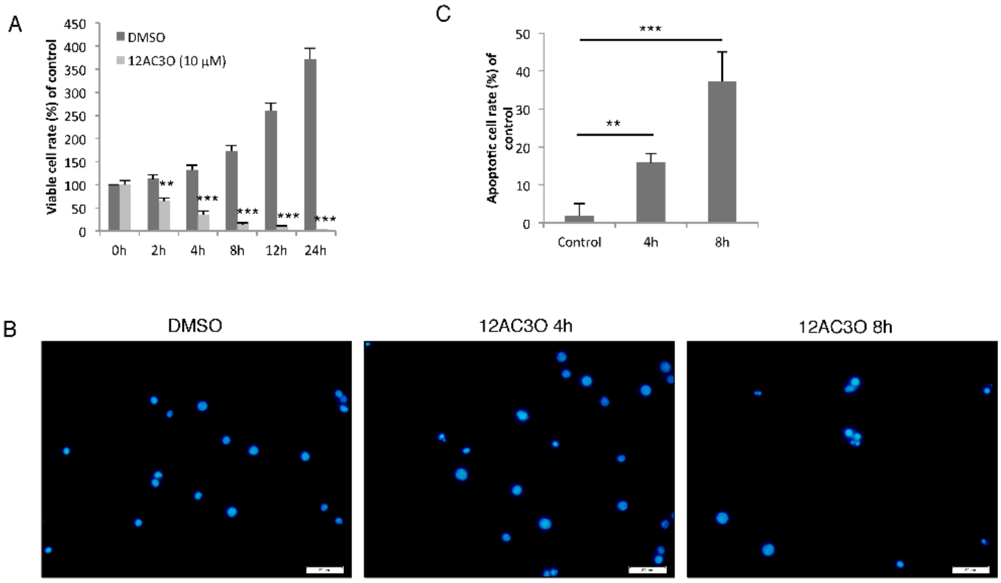

Figure S1. 12AC3O induced apoptosis in Jurkat T-cells as estimated by morphological examination. (**A**) Viable cell ratio of 12AC3O (10 μM)-treated Jurkat cells up to 24 h after the start of treatment. The viable cells were counted over time by using trypan-blue staining. *t*-test ** $p < 0.01$, *** $p < 0.001$ *versus* the control; (**B**,**C**) The morphological characteristics of apoptosis in Jurkat cells were observed by fluorescence microscopy using Hoechst 33342 staining (5 μg/μL). Bar is 50 μm. *t*-test ** $p < 0.01$, *** $p < 0.001$ *versus* the control.
